# Feasibility of low-dose dexmedetomidine for prevention of postoperative delirium after intracranial operations: a pilot randomized controlled trial

**DOI:** 10.1186/s12883-021-02506-z

**Published:** 2021-12-04

**Authors:** Xuan He, Kun-Ming Cheng, Yu-Qing Duan, Shan-Shan Xu, Hao-Ran Gao, Ming-Yue Miao, Hong-Liang Li, Kai Chen, Yan-Lin Yang, Linlin Zhang, Hong-Qiu Gu, Jian-Xin Zhou

**Affiliations:** 1 Department of Critical Care Medicine, Beijing Tiantan Hospital, Capital Medical University, Beijing, 100070 China; 2 Weifang Medical University, Weifang, 261041 Shandong China; 3 Clinical Trial and Research Center, Beijing Tiantan Hospital, Capital Medical University, Beijing, 100070 China

**Keywords:** Delirium, Postoperative, Intracranial operation, Prevention, Dexmedetomidine

## Abstract

**Background:**

Clinical trials have shown that dexmedetomidine might decrease the occurrence of postoperative delirium after major surgery, but neurosurgical patients were excluded from these studies. We aimed to determine the feasibility of conducting a full-scale randomized controlled trial of the effect of prophylactic low-dose dexmedetomidine on postoperative delirium in patients after elective intracranial operation for brain tumors.

**Methods:**

In this single-center, parallel-arm pilot randomized controlled trial, adult patients who underwent an elective intracranial operation for brain tumors were recruited. Dexmedetomidine (0.1 μg/kg/hour) or placebo was continuously infused from intensive care unit (ICU) admission on the day of surgery until 08:00 AM on postoperative day one. Adverse events during the study-drug administration were recorded. The primary feasibility endpoint was the occurrence of study-drug interruption. Delirium was assessed twice daily with the Confusion Assessment Method for the ICU during the first five postoperative days. The assessable rate of delirium evaluation was documented.

**Results:**

Sixty participants were randomly assigned to receive either dexmedetomidine (*n* = 30) or placebo (n = 30). The study-drug was stopped in two patients (6.7%) in the placebo group due to desaturation after new-onset unconsciousness and an unplanned reoperation for hematoma evacuation and in one patient (3.3%) in the dexmedetomidine group due to unplanned discharge from the ICU. The absolute difference (95% confidence interval) of study-drug interruption between the two groups was 3.3% (− 18.6 to 12.0%), with a noninferiority *P* value of 0.009. During the study-drug infusion, no bradycardia occurred, and hypotension occurred in one patient (3.3%) in the dexmedetomidine group. Dexmedetomidine tended to decrease the incidence of tachycardia (10.0% vs. 23.3%) and hypertension (3.3% vs. 23.3%). Respiratory depression, desaturation, and unconsciousness occurred in the same patient with study-drug interruption in the placebo group (3.3%). Delirium was evaluated 600 times, of which 590 (98.3%) attempts were assessable except in one patient in the placebo group who remained in a coma after an unplanned reoperation.

**Conclusions:**

The low rate of study-drug interruption and high assessable rate of delirium evaluation supported a fully powered trial to determine the effectiveness of low-dose dexmedetomidine on postoperative delirium in patients after intracranial operation for brain tumors.

**Trial registration:**

The trial was registered at ClinicalTrials.gov (NCT04494828) on 31/07/2020.

## Background

As one of the common complications after major surgery, postoperative delirium is associated with undesirable consequences, including higher morbidity and mortality, increased cost of care, and impairment of long-term quality of life after discharge [1, 2]. Prevention of postoperative delirium is recommended in the clinical guidelines and consensus statements [3–5]. However, neurosurgical patients are often excluded from interventional studies on postoperative delirium mainly due to potential impairment of consciousness and cognition due to their underlying condition [6–8].

Recent studies have shown that postoperative delirium is not uncommon after intracranial operations [9]. In four published cohort studies including a total of 2649 patients after brain tumor resection, postoperative delirium was diagnosed in 317 (12.0, 95% confidence interval: 10.8–13.3%) with an incidence ranging from 4.2 to 18.4% [10–13]. These studies also found that postoperative delirium was likely associated with a prolonged stay in the intensive care unit (ICU) and hospital [10, 12, 13], a higher incidence of nondelirium postoperative complications [13], a greater risk of an unfavorable functional outcome [10, 11], and higher hospital costs [12, 13]. Based on the prevalence and the potential association with adverse consequences for postoperative delirium in patients after intracranial operations for brain tumors, intervention studies are warranted.

Dexmedetomidine, a highly selective α_2_-adrenergic receptor agonist, has been investigated as a pharmacological intervention to prevent postoperative delirium [7, 8]. However, a higher incidence of hemodynamic adverse events was also identified in the dexmedetomidine group than in the control group in these studies [8]. Although low-dose dexmedetomidine without loading was employed in recent trials to minimize adverse events, bradycardia and hypotension were also observed in these studies [14–16]. The beneficial effects of dexmedetomidine may be offset by these adverse effects. Additionally, some adverse effects with distinct signs, such as bradycardia, may potentially result in unmasking during the conduction of the trial.

We performed this pilot randomized controlled trial with the aim of investigating the feasibility of low-dose dexmedetomidine infusion in the early postoperative period in patients after an intracranial operation for brain tumors. We hypothesized that the occurrence of study-drug interruption in the dexmedetomidine group would not be higher than that in the placebo group. We also investigated the adverse events that might potentially unmask the study-drug assignment, as well as the assessable rate of delirium evaluation in this population.

## Methods

### Study design and ethics

This single-center pilot trial with a randomized, double-blind, two parallel-arm, placebo-controlled design was approved by the Institutional Review Board of Beijing Tiantan Hospital, Capital Medical University (No. KY2019–091-02). All methods were performed in accordance with the Declaration of Helsinki. The trial was registered at ClinicalTrials.gov on 31/07/2020 (NCT04494828) and reported according to the Consolidated Standards of Reporting Trials (CONSORT) statement extension for pilot and feasibility trials. We obtained the written informed consents from the patients or their legal representatives. The present study was conducted in a 20-bed ICU at an academic affiliated hospital.

### Participants

All patients who underwent an elective intracranial procedure for cerebral tumors under general anesthesia and then were admitted to the ICU directly from the operating room or post-anesthesia care unit were screened by three qualified investigators (HLL, KC, and YLY). The exclusion criteria included [13]: (1) age under 18 years; (2) admission to the ICU after 10:00 PM; (3) medical records documented a preoperative history of mental or cognitive disorders, including schizophrenia, epilepsy, Parkinsonism, or dementia; (4) medical records documented an inability to communicate in the preoperative period due to coma or a language barrier; (5) history of drug abuse of psychoactive and anesthetic drugs; (6) known preoperative severe bradycardia (lower than 50 beats/min), sick sinus syndrome, second- or third-degree atrioventricular block, or left ventricular ejection fraction lower than 30%; (7) serious hepatic dysfunction defined as Child-Pugh class C; (8) severe renal dysfunction requiring renal replacement therapy before the surgery; (9) allergies to ingredients or components of dexmedetomidine hydrochloride; (10) American Society of Anesthesiologists classification of IV to VI; (11) moribund condition with a low likelihood of survival for more than 24 h; (12) pregnancy or lactating women; (13) enrolled in another clinical trial; or (14) refusal to participate.

After enrollment, demographic data, preoperative comorbidity, diagnosis on hospital admission, and perioperative information were collected.

### Randomization and blinding

Simple randomization at a 1:1 ratio was conducted using a computerized random digits table. The results were sealed in numbered opaque envelopes. The study-drugs (dexmedetomidine hydrochloride 200 μg/2 ml or normal saline 2 ml) were packed as clear aqueous solutions with the same characteristics in the same type of 3-ml ampoules. Jiangsu Nhwa Pharmaceutical Co., Ltd. (Jiangsu, China) manufactured and provided the study-drugs. Prior to the study, a pharmacist unenrolled in the rest of the study encoded ampoules according to the randomization results.

Consecutively recruited patients were randomly assigned to the dexmedetomidine group (receive dexmedetomidine hydrochloride) or the placebo group (receive normal saline). The study coordinator (LZ) distributed the study-drugs. The patients were unaware of their group allocation, as were the ICU physicians and other healthcare providers who were responsible for patient care, and the investigators who performed data collection, follow-up and data analysis.

### Study-drug administration

The study-drug was diluted with normal saline to 50 ml and intravenously infused at a rate of 0.025 ml/kg/hour. This represented an infusion rate of 0.1 μg/kg/hour dexmedetomidine in the dexmedetomidine group.

The intravenous infusion was started immediately after enrollment on the day of the operation and continued until 08:00 AM on postoperative day one. During the study, open-labeled dexmedetomidine was not allowed. Scopolamine and penehyclidine were prohibited. Atropine could only be administered to treat bradycardia.

During the study, apart from the administration of the study-drugs, the care of the patients was decided by the responsible ICU physicians according to the clinical routine in our department.

### Routine management of pain, agitation and delirium

During the study, pain, agitation and delirium were managed according to the recommendations in guidelines proposed by the European Society of Anaesthesiology and the American Society of Critical Care Medicine [3, 17], which have been employed as routine clinical strategies in our ICU [13, 18, 19].

Postoperative analgesia was routinely administered along with patient-controlled intravenous analgesia (PCIA), which was comprised of sufentanil 100 mg and tropisetron 10 mg in 100 ml of 0.9% NaCl solution. A basal PCIA infusion (2 ml/hour) was started after confirmation of the patient’s cardiorespiratory stability and the recovery of consciousness [13, 18, 19]. Pain assessment was performed every 6 h or as needed using the numeric rating scale (NRS) or the Critical-Care Pain Observation Tool [20]. Remifentanil or butorphanol was used in patients who required analgesia. Agitation-sedation assessment was also performed every 6 h or as needed using the Richmond Agitation-Sedation Scale (RASS) [21]. Propofol or midazolam was administered to patients who exhibited agitation, and a light sedation depth was maintained with a RASS score of − 2 to + 1. Delirium was assessed twice daily using the Confusion Assessment Method for the ICU (CAM-ICU), which was validated in mechanically ventilated patients and nonintubated ICU patients [22, 23]. The Chinese version of the CAM-ICU was validated in the ICU setting in mainland China [24], and its feasibility had been previously established in studies reported by our group and others [13, 15]. In patients developing delirium, nonpharmacological treatments were first used, mainly including repeated reorientation, early mobilization and hearing aids. Haloperidol was only administered to patients with hyperactive delirium and severe agitation.

### Adverse events and management

Adverse events were monitored from the start of study drug infusion until ICU discharge or 24 h, whichever came first. Predicted adverse events related to the use of dexmedetomidine included bradycardia, hypotension, respiratory depression, and desaturation [14, 15, 25]. Bradycardia was defined as a heart rate lower than 50 beats/min or a decrease of more than 20% from baseline (before the study-drug infusion) in cases of a baseline value less than 63 beats/min [14, 15, 25]. Hypotension was defined as systolic blood pressure lower than 90 mmHg or a decrease of more than 20% from baseline in cases of a baseline value less than 113 mmHg [14, 15, 25]. Respiratory depression was defined as arterial partial pressure of carbon dioxide greater than 50 mmHg or respiratory rate less than 10 breaths/min [14, 15, 25]. Desaturation was defined as pulse oxygen saturation lower than 90% or a decrease of more than 5% of the absolute value from baseline [14, 15, 25]. Tachycardia and hypertension were also recorded. Tachycardia was defined as a heart rate greater than 120 beats/min or an increase of more than 20% from baseline in cases of a baseline value greater than 100 beats/min [14, 15, 25]. Hypertension was defined as systolic blood pressure greater than 160 mmHg or an increase of more than 20% from baseline in cases of a baseline value greater than 133 mmHg [14, 15, 25]. Unconsciousness was documented as a Glasgow Coma Scale (GCS) score less than 9 [13, 18, 19].

Intervention for hypotension included fluid resuscitation and/or administration of medication. Bradycardia, tachycardia, and hypertension were treated with medication. Intervention for respiratory depression and desaturation included oxygen administration, physical therapy, endotracheal intubation, and/or mechanical ventilation. In cases of new-onset unconsciousness, physical examination and/or computed tomography were performed, and a neurosurgeon was consulted. The treatment of adverse events was determined by the responsible ICU physicians, who could decrease or stop the study-drug infusion if necessary.

The ICU physicians could also request unmasking of blinding when treatment failure or other conditions were deemed as making it necessary. Because each ampoule containing dexmedetomidine or placebo had a unique randomization number, urgent unmasking would not reveal the group allocations of the other enrolled patients.

### Data collection and endpoints

Before the initiation of the trial, four clinical research fellows (YQD, SSX, HRG, and MYM) who were not involved in the care of the patients were trained to follow the study protocol and were responsible for data collection and follow-up. They were also trained to perform the CAM-ICU evaluation by an expert from the Department of Psychiatry as we reported previously [13]. The CAM-ICU assessment was performed in two steps [22, 23]. The arousal level was first assessed by RASS [21]. If the patient was not responsive to verbal stimuli (i.e., RASS score ≤ − 4), the remaining delirium assessment was aborted, and the patient was recorded as comatose. When the RASS score was greater than or equal to − 3, delirium was evaluated using the CAM-ICU. The CAM-ICU consists of four key features: (1) acute onset of a change in mental status or a fluctuating level of consciousness; (2) inattention; (3) disorganized thinking; and (4) an altered level of consciousness [22–24]. Delirium was diagnosed when the patient displayed the first and second features, plus either the third or fourth feature.

After the stop of study-drug infusion at 08:00 AM on postoperative day one, vital signs before the study-drug infusion and one hour after the infusion was started were downloaded from the monitor.

In case of study-drug interruption, the causes which might include adverse events, unplanned reoperation, the responsible physician identifying other conditions, or refusal of continuing use by the patients or their legal representatives, were documented in the case report form.

The patients were followed up twice daily (08:00 AM to 10:00 AM and 06:00 PM to 08:00 PM) during the first five postoperative days and then weekly until hospital discharge or until 28 days after the operation. Postoperative delirium was defined as positive CAM-ICU in the first five postoperative days [3].

The primary endpoint was the occurrence of study-drug interruption, which represented the feasibility of prophylactic use of low-dose dexmedetomidine.

Secondary endpoints included: (1) assessable rate of delirium; (2) duration of study-drug infusion; (3) the use of sedatives and analgesics during the study-drug infusion; (4) RASS, pain intensity evaluated using the NRS, and subjective sleep quality evaluated using the NRS with an 11-point scale [26] on the morning of postoperative day one; (5) time to extubation; (6) incidence of postoperative delirium during the first five postoperative days; (7) length of stay in the ICU and hospital after the operation; (8) incidence of nondelirium complications, which were defined as conditions needing interventions; (9) cognitive impairment evaluated using the Mini-Cog at the end of follow-up [27]; and (10) all-cause hospital mortality.

### Statistical analysis

We selected study-drug interruption as the primary endpoint to demonstrate the feasibility of low-dose dexmedetomidine infusion in the early postoperative period. Two studies compared low-dose dexmedetomidine (0.1 μg/kg/hour without a loading infusion) with placebo in elderly patients after noncardiac surgery, and the rate of study-drug interruption was 9.1 to 10.5% in the dexmedetomidine group and 2.6 to 4.6% in the placebo group [14, 15]. We assumed that the study-drug interruption rate would be 4% in the placebo group, and set the noninferiority margin at 15%, alpha at 0.05, and statistical power at 0.90. Thirty patients per group were needed to demonstrate noninferiority of dexmedetomidine without consideration of dropout [28]. Farrington-Manning test was used to assess the noninferiority of dexmedetomidine to placebo on the rate of study-drug interruption.

Categorical variables are presented as numbers and percentages and were analyzed by the χ^2^-test or Fisher’s exact test. Continuous variables were checked for a normal distribution and presented as the mean and standard deviation or median and interquartile range as appropriate. Comparison of continuous variables was performed by Student’s t-test for normally distributed variables and the Mann-Whitney U test for nonnormally distributed variables.

Statistical analysis was performed using SPSS 20.0 (SPSS Inc., Chicago, IL, USA). A *P* value of less than 0.05 was considered statistically significant.

## Results

**Fig. 1:**
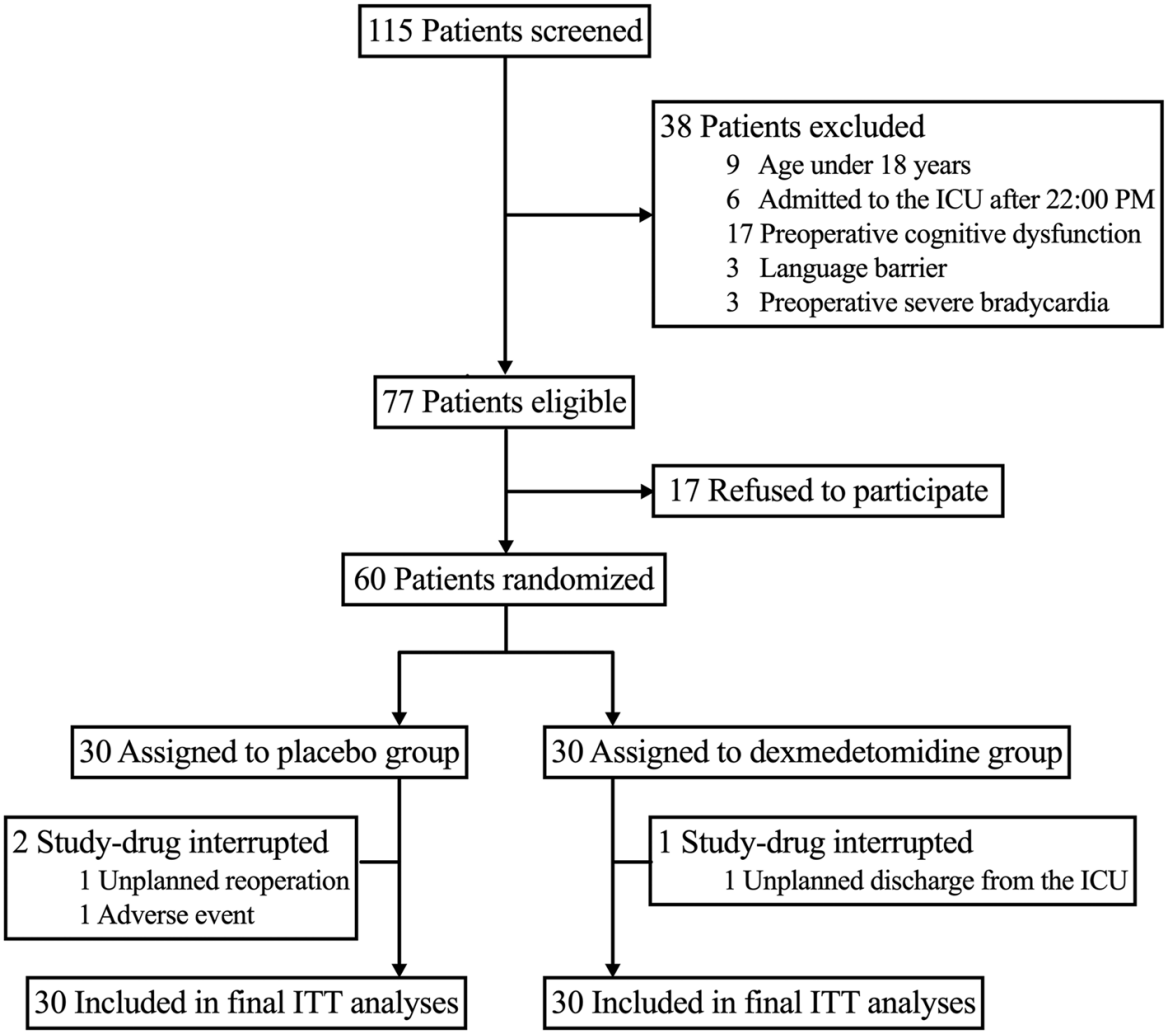
Flowchart of the trial. ITT intention to treat

Between August 12 and December 12, 2020, 115 patients were screened for study eligibility, of whom 60 patients were enrolled and randomly assigned to receive either dexmedetomidine (*n* = 30) or placebo (n = 30) (Fig. 1). Baseline characteristics and perioperative data before the study-drug administration are shown in Table 1. Overall, the two groups were well matched except that a higher incidence of emergence delirium (13.3% vs. 0.0%) was found in the placebo group. The time intervals from the end of the operation to study-drug infusion were 5.8 ± 2.0 and 5.5 ± 2.1 h in the placebo group and the dexmedetomidine group, respectively.

**Table 1:** Baseline and perioperative data before the study-drug administration

	Placebo (*n* = 30)	Dexmedetomidine (n = 30)	*P*
Age, mean ± SD, years	48 ± 11	51 ± 11	0.204
Male, n (%)	15 (50.0)	13 (43.3)	0.605
Body mass index, mean ± SD, kg/m^2^	23.8 ± 3.8	25.2 ± 4.1	0.165
Education, median (IQR), years	15 (7–16)	12 (7–15)	0.521
Preoperative comorbidity, n (%)			
Hypertension	6 (20.0)	6 (20.0)	> 0.999
Coronary heart disease	1 (3.3)	0 (0.0)	> 0.999
Diabetes mellitus	1 (3.3)	2 (6.7)	> 0.999
Ischemic stroke	1 (3.3)	0 (0.0)	> 0.999
History of smoking,* n (%)	10 (33.3)	9 (30.0)	0.781
Alcoholism,† n (%)	4 (13.3)	4 (13.3)	> 0.999
Preoperative ASA classification, n (%)			0.185
I	0 (0.0)	2 (6.7)	
II	28 (93.3)	27 (90.0)	
III	2 (6.7)	1 (3.3)	
Duration of anesthesia, mean ± SD, hours	6.5 ± 1.8	6.5 ± 1.7	0.979
Medication during anesthesia, n (%)			
Midazolam	16 (53.3)	20 (66.7)	0.292
Propofol	30 (100.0)	30 (100.0)	> 0.999
Etomidate	13 (43.3)	10 (33.3)	0.426
Dexmedetomidine	4 (13.3)	1 (3.3)	0.353
Sufentanil	30 (100.0)	30 (100.0)	> 0.999
Remifentanil	28 (93.3)	27 (90.0)	0.640
Sevoflurane	26 (86.7)	28 (93.3)	0.389
Desflurane	1 (3.3)	0 (0.0)	> 0.999
Glucocorticoids	1 (3.3)	1 (3.3)	> 0.999
Atropine	8 (26.7)	7 (23.3)	0.766
Penehyclidine Hydrochloride	9 (30.0)	15 (50.0)	0.114
Mannitol	9 (30.0)	12 (40.0)	0.417
Duration of operation, mean ± SD, hours	4.7 ± 1.4	4.9 ± 1.6	0.775
Body position, n (%)			0.190
Supine	15 (50.0)	10 (33.3)	
Lateral	15 (50.0)	20 (66.7)	
Frontal approach of operation, n (%)	10 (33.3)	9 (30.0)	0.781
Location of the tumor, n (%)			0.289
Supratentorial	14 (46.7)	9 (30.0)	
Infratentorial	11 (36.7)	17 (56.7)	
Others‡	5 (16.6)	4 (13.3)	
Malignant tumor, n (%)	7 (23.3)	6 (20.0)	0.754
Total intraoperative infusion, median (IQR), ml	2600 (2463–3138)	2600 (2250–3513)	0.715
Estimated intraoperative bleeding, median (IQR), ml	200 (200–362)	225 (200–425)	0.464
Blood transfusion during operation, n (%)	4 (13.3)	7 (23.3)	0.317
Episode of intraoperative hypotension, n (%)	6 (20.0)	8 (26.7)	0.761
APACHE II score on ICU admission, median (IQR)	10 (7–12)	10 (8–12)	0.316
GCS on ICU admission, median (IQR)	14 (14–14)	14 (10–14)	0.334
Endotracheal intubation on ICU admission, n (%)	4 (13.3)	8 (26.7)	0.210
Patient-controlled intravenous analgesia, n (%)	12 (40.0)	11 (36.7)	0.791
Emergence delirium before study drug infusion, n (%)	4 (13.3)	0 (0.0)	0.038
Time interval between the end of operation and study drug infusion, mean ± SD, hours	5.5 ± 2.1	5.8 ± 2.0	0.613

* Smoking half a pack of cigarettes per day for at least 2 years. † Two drinks or more daily or weekly consumption of the equivalent of 150 ml of alcohol. ‡ Including saddle area, the tumor across the supratentorial and infratentorial area, and the boundary of the tumor is not clear

APACHE Acute Physiologic Assessment and Chronic Health Evaluation, ASA American Society of Anesthesiologists, GCS Glasgow Coma Scale, ICU intensive care unit, IQR, interquartile range

No significant difference was found in the duration of study-drug infusion between the two groups (10.3 [9.9–11.5] vs. 10.7 [10.2–11.4] hours, *P* = 0.387). Study-drug interruption occurred in three patients (5.0%). The study-drug was stopped in two patients (6.7%) in the placebo group, in one patient because of respiratory depression and desaturation due to new-onset unconsciousness, and in another patient because of an unplanned reoperation for hematoma evacuation. In one patient in the dexmedetomidine group (3.3%), the study-drug was stopped due to unplanned discharge from the ICU. The absolute difference (95% confidence interval) of study-drug interruption between the two groups was 3.3% (− 18.6 to 12.0%), with a noninferiority test *P* value of 0.009. No unmasking of allocation was requested during the study.

**Fig. 2:**
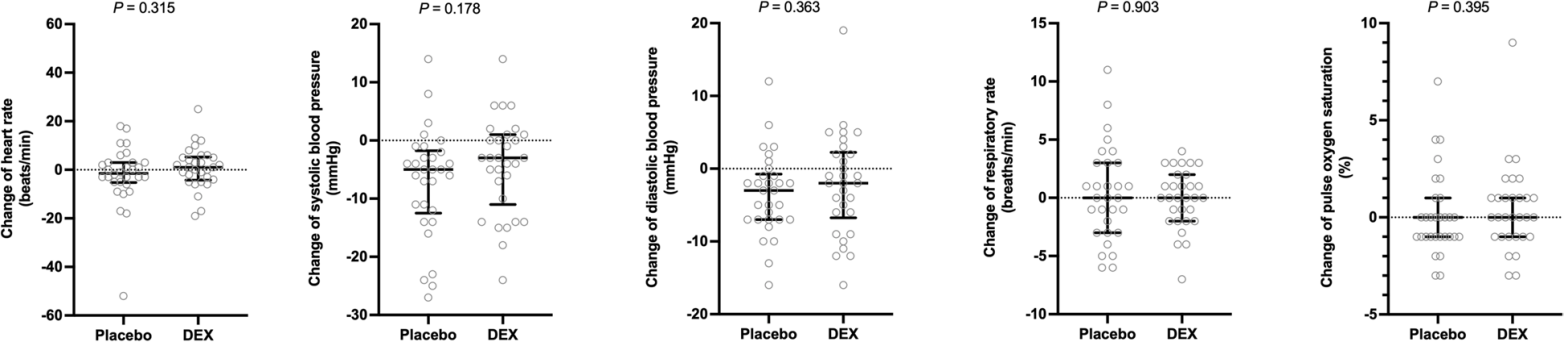
Changes of vital signs before and at one hour after the study-drug infusion (after minus before). Individual data, the median and interquartile range are shown. DEX dexmedetomidine

Changes in heart rate, systolic and diastolic blood pressure, respiratory rate, and pulse oxygen saturation before and at one hour after the study-drug infusion (after minus before) are shown in Fig. 2. No significant differences were found in the changes in these vital signs (*P* values ranged from 0.178 to 0.903).

The incidence of adverse events during the study drug administration is summarized in Table 2. No bradycardia occurred during the study. Hypotension occurred in one patient (3.3%) in the dexmedetomidine group. Compared to the dexmedetomidine group, the incidences of tachycardia (23.3% vs. 10.0%) and hypertension (23.3% vs. 3.3%) were higher in the placebo group, but the difference was not statistically significant (*P* = 0.299 and 0.052). In the placebo group, respiratory depression and desaturation occurred in one case due to unconsciousness. The study-drug was stopped immediately. After back percussion, suctioning, and manual ventilation via an oxygen mask, the patient’s respiration recovered, and the pulse oxygen saturation increased to 97%. The patient then regained consciousness. A CT scan showed a normal postoperative condition.

**Table 2:** Adverse events

	Placebo (*n* = 30)	Dexmedetomidine (*n* = 30)	*P*
Bradycardia, n (%)	0 (0.0)	0 (0.0)	> 0.999
Bradycardia with intervention, n (%)	0 (0.0)	0 (0.0)	> 0.999
Hypotension, n (%)	0 (0.0)	1 (3.3)	> 0.999
Hypotension with intervention, n (%)	0 (0.0)	1 (3.3)	> 0.999
Tachycardia, n (%)	7 (23.3)	3 (10.0)	0.299
Tachycardia with intervention, n (%)	4 (13.3)	2 (6.7)	0.671
Hypertension, n (%)	7 (23.3)	1 (3.3)	0.052
Hypertension with intervention, n (%)	5 (16.7)	1 (3.3)	0.159
Respiratory depression, n (%)	4 (13.3)	3 (10.0)	> 0.999
Respiratory depression with intervention, n (%)*	1 (3.3)	0 (0.0)	> 0.999
Desaturation, n (%)	2 (6.7)	1 (3.3)	> 0.999
Desaturation with intervention, n (%)*	1 (3.3)	0 (0.0)	> 0.999
Unconsciousness, n (%)	1 (3.3)	0 (0.0)	> 0.999
Unconsciousness with intervention, n (%)*	1 (3.3)	0 (0.0)	> 0.999

* Desaturation occurred in one case due to unconsciousness

**Fig. 3:**
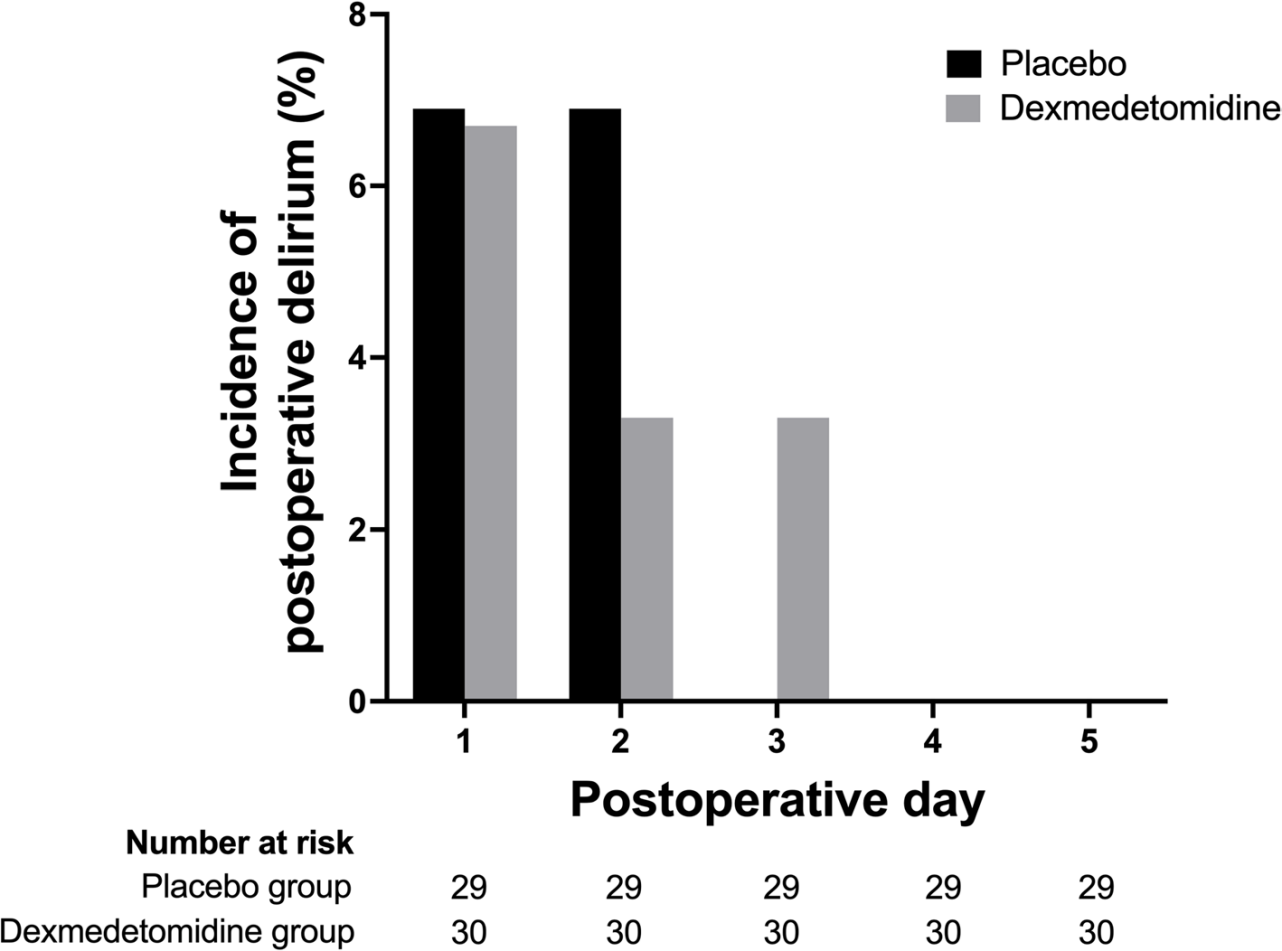
Daily prevalence of postoperative delirium. In the placebo group, delirium could not be assessed in one patient who remained coma after unplanned reoperation for hematoma evacuation

No patient was discharged from the hospital during the first five postoperative days. CAM-ICU was evaluated in 60 patients for 600 times (twice daily for five days), of which 590 attempts (98.3%) were assessable, except in one patient in the placebo group who remained in a coma after an unplanned reoperation for hematoma evacuation. Table 3 shows the RASS evaluation during the first five postoperative days. No significant difference was found in RASS scores between the two groups and across time points. No significant difference was found in the incidence of postoperative delirium between the two groups (3/29, 10.3% in the placebo group vs. 2/30, 6.7% in the dexmedetomidine group, *P* = 0.671), with all postoperative delirium cases occurring within the first three postoperative days (Fig. 3).

**Table 3:** RASS scores during the first five postoperative days

Postoperative days	Placebo (n = 30)	Dexmedetomidine (n = 30)
1-AM	0 (0–0) [−5–0]	0 (0–0) [−1–0]
1-PM	0 (0–0) [−5–1]	0 (0–0) [−2–1]
2-AM	0 (0–0) [− 5–2]	0 (0–0) [−3–0]
2-PM	0 (0–0) [−5–1]	0 (0–0) [− 2–0]
3-AM	0 (0–0) [−5–0]	0 (0–0) [−1–0]
3-PM	0 (0–0) [−5–0]	0 (0–0) [−3–0]
4-AM	0 (0–0) [−5–0]	0 (0–0) [0–0]
4-PM	0 (0–0) [−5–0]	0 (0–0) [0–0]
5-AM	0 (0–0) [−5–0]	0 (0–0) [0–0]
5-PM	0 (0–0) [−5–0]	0 (0–0) [0–0]

Data are shown as median (interquartile range) [minimum–maximum]. No significant difference was found in RASS score between the two groups and across time points

Clinical outcome variables are shown in Table 4. No significant differences were found in the use of sedatives and opioids during the study-drug infusion or in the RASS and subjective pain intensity at the end of study-drug infusion between the two groups. The subjective sleep quality score was lower in the dexmedetomidine group than in the placebo group (2 [1–7] vs. 5 [2–9﻿]), but the difference was not statistically significant (*P* = 0.142). No significant differences were found in any other clinical outcomes between the two groups. No patient died within 28 days after the operation.

**Table 4:** Clinical outcome variables

	Placebo (n = 30)	Dexmedetomidine (*n* = 30)	*P*
Use of sedatives during study-drug infusion, n (%)	1 (3.3)	3 (10.0)	0.601
Propofol	0 (0.0)	1 (3.3)	
Midazolam	1 (3.3)	2 (6.7)	
Use of opioids during study-drug infusion, n (%)	6 (20.0)	5 (16.7)	> 0.999
Remifentanil	1 (3.3)	1 (3.3)	
Butorphanol	5 (16.7)	4 (13.3)	> 0.999
RASS at the end of study-drug infusion, median (IQR)	0 (0–0)	0 (0–0)	> 0.999
Subjective pain score on postoperative day one, median (IQR)	4 (0–6)	3 (2–4)	0.192
Subjective sleep score on postoperative day one, median (IQR)	5 (2–9)	2 (1–7)	0.142
Time to extubation,* median (IQR), hours	17 (16–19)	18 (15–19)	> 0.999
Non-delirium complications, n (%)	7 (23.3)	4 (13.3)	0.506
Length of stay in the ICU, median (IQR), hours	17 (15–19)	18 (16–19)	0.376
Length of stay in hospital after the operation, median (IQR), days	9 (8–12)	11 (8–15)	0.247
Mini-Cog at the end of follow-up, median (IQR)	5 (4–5)	5 (4–5)	0.507

* Result in endotracheal intubated patients on the ICU admission

ICU intensive care unit, IQR interquartile range

## Discussion

This pilot randomized trial examined the feasibility of low-dose dexmedetomidine for the prevention of postoperative delirium after elective intracranial operations for brain tumors. Dexmedetomidine was noninferior to placebo on the rate of study-drug interruption. No unmasking was requested during the study. No significant differences were found between the two groups in changes in vital signs after the study-drug infusion. The incidences of adverse events did not differ between the two groups. Delirium was assessable in the majority of patients.

Before the initiation of a formal clinical trial for determining the efficacy of dexmedetomidine on the prevention of postoperative delirium in patients undergoing intracranial surgery, two questions should be answered. First, although the safety of low-dose dexmedetomidine has been reported in patients following noncardiac surgery [15, 16], is this dosage regimen tolerated in patients after intracranial operations? Second, although the CAM-ICU has been used for delirium assessment in patients admitted to the ICU after nonneurosurgical operations [15, 16], can this instrument be utilized for postoperative neurosurgical patients receiving dexmedetomidine? Therefore, we performed this pilot trial aiming to answer these two questions and to provide basic data for further interventional trials.

Although the use of a sedative dose of dexmedetomidine (0.2–1.4 μg/kg/hour, with or without a loading dose) in critically ill patients is likely associated with a reduced risk of delirium, higher incidences of bradycardia and hypotension are also found [29, 30]. To obtain the effect of postoperative delirium prevention and simultaneously avoid adverse events, low-dose dexmedetomidine was investigated in patients after surgery [7, 8]. In a pilot study primarily evaluating the effect of dexmedetomidine on sleep quality, Wu et al. randomly assigned 76 ICU-admitted patients older than 65 years undergoing noncardiac surgery to receive a continuous infusion of dexmedetomidine at a rate of 0.1 μg/kg/hour for 15 h or placebo [14]. A higher incidence of hypotension was found in the dexmedetomidine group but without any need for intervention. In two other large-sample trials, low-dose dexmedetomidine (0.1 μg/kg/hour, without loading) was administered to elderly patients after noncardiac surgery who were admitted to the ICU [15] or the general surgical wards [16]. The incidence of bradycardia and hypotension did not differ between the dexmedetomidine group and the placebo group. Neurosurgical patients were excluded from all three trials [14–16]. In our previous randomized controlled trial in patients with delayed extubation after elective craniotomy, a sedative dose of dexmedetomidine (0.6 μg/kg/hour without loading) was continuously infused until 30 min after extubation or 24 h [25]. Significantly higher incidences of bradycardia (5.3%) and hypotension (8.0%) were found in the dexmedetomidine group. Dexmedetomidine was urgently discontinued in 5.3 and 4.0% of patients because of bradycardia and hypotension, respectively. In the present study, no bradycardia occurred, and hypotension was only found in one case during low-dose dexmedetomidine administration. This prevalence is much lower than previous reports in noncardiac elderly patients (9.3–31.6% for bradycardia [14–16] and 32.6–39.5% for hypotension [14, 15]). We enrolled relatively young patients with fewer preoperative comorbidities, which may explain the discrepancy in the results. In accordance with previous reports, the incidences of tachycardia and hypertension were decreased in the dexmedetomidine group, which might suggest a hemodynamic stability effect of dexmedetomidine [15]. The study-drug was interrupted in only one case in the dexmedetomidine group due to unplanned discharge from the ICU but no cases were interrupted due to adverse events. Our results suggested that a low-dose regimen of dexmedetomidine could be feasibly administered to patients after elective intracranial operations.

An analysis based on a large national data registry program showed that approximately 3% of patients underwent unplanned reoperation after brain tumor resection, with the most common reason being intracranial hematoma evacuation [31]. These patients usually remain in a coma after the reoperation, resulting in difficulty in delirium assessment. In the present trial, the study drug was stopped in one case in the placebo group due to an unplanned reoperation. The CAM-ICU was not assessable during the first five postoperative days because the patient remained in a coma after the reoperation. In another case in the dexmedetomidine group, the study-drug was stopped because of unplanned ICU discharge due to a limitation of ICU bed resources. The influence of these types of factors on the feasibility of the protocol should also be considered during the design and sample size calculation of future research.

Apart from study-drug interruption due to adverse events, a risk of unblinding exists due to cardiovascular responses to dexmedetomidine. This may potentially influence the accuracy of delirium evaluation because of the subjective nature of the assessment instruments, such as the CAM-ICU. The change in heart rate after the study drug administration did not differ between the two groups, with values distributed around zero (Fig. 2). No changes of other vital signs differed between the groups. This may be due to the very low dose used in the present study. These results suggested the feasibility of blinding during the infusion of low-dose dexmedetomidine.

One major feasibility consideration for studies of delirium in neurosurgical patients is selection of the evaluation instrument. The CAM-ICU and the Intensive Care Delirium Screening Checklist are recommended for delirium assessment in ICU patients [17]. For patients undergoing major surgery admitted to the ICU, the CAM-ICU is also recommended for postoperative delirium assessment by the European Society of Anaesthesiology in postoperative patients [3]. No consensus has been achieved for delirium assessment tools in patients with neurological disorders, including patients after intracranial operations. A systematic review and meta-analysis revealed that the sensitivity and specificity of the CAM-ICU to screen for delirium in neurocritically ill patients referenced against the Diagnostic and Statistical Manual of Mental Disorders (Fourth Edition) ranged from 62 to 76% (median 69%) and 74–98% (median 77%), respectively [32]. The main concern about the use of the CAM-ICU in neurocritically ill patients is whether this instrument is assessable. To date, three cohort studies have used the CAM-ICU for postoperative delirium diagnosis in patients after brain tumor resection [11–13], two of which did not report the rate of “not assessable” evaluations [11, 12]. In our previous study enrolling 815 patients after intracranial operations, CAM-ICU evaluation was attempted twice daily on postoperative day one and three, of which 27 (3.3%) and 20 (2.5%) were marked as “not assessable” [13]. Finally, 15 (1.8%) patients were excluded because the CAM-ICU was not able to assess them on postoperative day one and three due to sensory and mixed aphasia in 9 patients, a coma in 4 patients, and hearing loss in 2 patients. In the present study, which included 60 patients after brain tumor resections, 600 CAM-ICU evaluations were attempted during the first five postoperative days. Ten (1.7%) attempts failed in one patient who remained in a coma after an unplanned reoperation. These proportions were all markedly lower than the reported unable-to-assess rates of CAM-ICU in mixed neurocritically ill cohorts (18–34%) [33, 34]. Our results suggested that the CAM-ICU could be a feasible instrument for use in delirium assessment in patients after elective intracranial operations.

Balancing between the treatment groups is also a factor influencing the feasibility of the study protocol. Previous studies have demonstrated that midazolam dominant sedation was associated with a high incidence of delirium [35]. Our data showed that the use of midazolam, either during the operation or during the study-drug infusion, was not significantly different between the two groups. Another potential factor relating to postoperative delirium is the emergence delirium [13]. The overall incidence of emergence delirium (4/60, 6.6%) in the present study was comparable to the result in the population excluding neurosurgical patients (4.7%) [36], but was much lower than our previous report in patients after intracranial operations (20.3%) [13]. The incidence of emergence delirium in the dexmedetomidine group (0%) was significantly lower than that in the placebo group (13.3%, *P* = 0.038) in the present study. This might be due to the possible effect of dexmedetomidine. However, the incidence of emergency delirium was not the primary endpoint in our pilot trial. Further study is needed to investigate the effect of dexmedetomidine on emergency delirium. Anyway, given the potential impact of emergence delirium on the occurrence of postoperative delirium, stratification of the enrolled patients might be a better solution.

There are limitations of the present trial. First, this study only enrolled patients admitted to the ICU after the operation. This population represents those at high risk of postoperative delirium [3–5]. Therefore, our results may be limited for generalization to all patients undergoing intracranial surgery. Second, we used the CAM-ICU to diagnose delirium in the present study because this is the recommended assessment tool for ICU patients and postoperative patients [3, 17]. A preliminary meta-analysis has shown that the CAM-ICU is assessable in neurocritically ill patients with acceptable sensitivity and specificity for delirium detection [32]. Additionally, large cohort studies have shown the feasibility of CAM-ICU for postoperative delirium evaluation in patients after intracranial tumor resection [11–13]. However, the accuracy and reliability of the CAM-ICU in the target population needs further investigation. Third, our primary endpoint was the rate of study-drug interruption during the study. It is underpowered to compare other outcomes, such as the incidence of postoperative delirium. These results should be interpreted with caution. Additionally, the impact of the study drug on the use of comedications and other managements as an indirect measure of the effect of dexmedetomidine should be included in the future full-powered trial.

## Conclusions

The low rate of study-drug interruption and high assessable rate of delirium evaluation suggest that the conduction of a fully powered trial seems feasible to investigate the efficacy of low-dose dexmedetomidine to prevent postoperative delirium in patients after elective intracranial operations for brain tumors.

## Data Availability

Data can be accessed by contacting the corresponding author on reasonable request.

## Abbreviations

APACHE: Acute Physiologic Assessment and Chronic Health Evaluation
ASA: American Society of Anesthesiologists
DEX: dexmedetomidine
GCS: Glasgow Coma Scale
ICU: intensive care unit
IQR: interquartile range
ITT: intention to treat
NRS: numeric rating scale
PCIA: patient-controlled intravenous analgesia
RASS: Richmond Agitation-Sedation Scale

## References

[1] Jin Z, Hu J, Ma D (2020). Postoperative delirium: perioperative assessment, risk reduction, and management. Br J Anaesth.

[2] Curtis MS, Forman NA, Donovan AL, Whitlock EL (2020). Postoperative delirium: why, what, and how to confront it at your institution. Curr Opin Anaesthesiol.

[3] Aldecoa C, Bettelli G, Bilotta F, Sanders RD, Audisio R, Borozdina A (2017). European Society of Anaesthesiology evidence-based and consensus-based guideline on postoperative delirium. Eur J Anaesthesiol.

[4] Mahanna-Gabrielli E, Schenning KJ, Eriksson LI, Browndyke JN, Wright CB, Culley DJ (2019). State of the clinical science of perioperative brain health: report from the American Society of Anesthesiologists Brain Health Initiative Summit 2018. Br J Anaesth.

[5] Hughes CG, Boncyk CS, Culley DJ, Fleisher LA, Leung JM, McDonagh DL (2020). Perioperative quality initiative (POQI) 6 workgroup: American Society for Enhanced Recovery and Perioperative Quality Initiative Joint Consensus Statement on postoperative delirium prevention. Anesth Analg.

[6] Igwe EO, Nealon J, Mohammed M, Hickey B, Chou KR, Chen KH (2020). Multi-disciplinary and pharmacological interventions to reduce post-operative delirium in elderly patients: a systematic review and meta-analysis. J Clin Anesth.

[7] Duan X, Coburn M, Rossaint R, Sanders RD, Waesberghe JV, Kowark A (2018). Efficacy of perioperative dexmedetomidine on postoperative delirium: systematic review and meta-analysis with trial sequential analysis of randomised controlled trials. Br J Anaesth.

[8] Shen QH, Li HF, Zhou XY, Yuan XZ (2020). Dexmedetomidine in the prevention of postoperative delirium in elderly patients following non-cardiac surgery: a systematic review and meta-analysis. Clin Exp Pharmacol Physiol.

[9] Viderman D, Brotfain E, Bilotta F, Zhumadilov A (2020). Risk factors and mechanisms of postoperative delirium after intracranial neurosurgical procedures. Asian J Anesthesiol.

[10] Flanigan PM, Jahangiri A, Weinstein D, Dayani F, Chandra A, Kanungo I (2018). Postoperative delirium in glioblastoma patients: risk factors and prognostic implications. Neurosurgery..

[11] Budėnas A, Tamašauskas Š, Šliaužys A, Navickaitė I, Sidaraitė M, Pranckevičienė A (2018). Incidence and clinical significance of postoperative delirium after brain tumor surgery. Acta Neurochir.

[12] Chen H, Jiang H, Chen B, Fan L, Shi W, Jin Y (2020). The incidence and predictors of postoperative delirium after brain tumor resection in adults: a cross-sectional survey. World Neurosurg.

[13] Wang CM, Huang HW, Wang YM, He X, Sun XM, Zhou YM (2020). Incidence and risk factors of postoperative delirium in patients admitted to the ICU after elective intracranial surgery: a prospective cohort study. Eur J Anaesthesiol.

[14] Wu XH, Cui F, Zhang C, Meng ZT, Wang DX, Ma J (2016). Low-dose dexmedetomidine improves sleep quality pattern in elderly patients after noncardiac surgery in the intensive care unit: a pilot randomized controlled trial. Anesthesiology..

[15] Su X, Meng ZT, Wu XH, Cui F, Li HL, Wang DX (2016). Dexmedetomidine for prevention of delirium in elderly patients after non-cardiac surgery: a randomised, double-blind, placebo-controlled trial. Lancet..

[16] Sun Y, Jiang M, Ji Y, Sun Y, Liu Y, Shen W (2019). Impact of postoperative dexmedetomidine infusion on incidence of delirium in elderly patients undergoing major elective noncardiac surgery: a randomized clinical trial. Drug Des Devel Ther.

[17] Devlin JW, Skrobik Y, Gelinas C, Needham DM, Slooter AJC, Pandharipande PP (2018). Clinical practice guidelines for the prevention and management of pain, agitation/sedation, delirium, immobility, and sleep disruption in adult patients in the ICU. Crit Care Med.

[18] Huang HW, Yan LM, Yang YL, He X, Sun XM, Wang YM (2018). Bi-frontal pneumocephalus is an independent risk factor for early postoperative agitation in adult patients admitted to intensive care unit after elective craniotomy for brain tumor: a prospective cohort study. PLoS One.

[19] Shan K, Cao W, Yuan Y, Hao JJ, Sun XM, He X, et al. Use of the critical-care pain observation tool and the bispectral index for the detection of pain in brain-injured patients undergoing mechanical ventilation: a STROBE-compliant observational study. Medicine (Baltimore). 2018;97:e10985. 10.1097/MD.0000000000010985 PMC6392730 29851854

[20] Gélinas C, Fillion L, Puntillo KA, Viens C, Fortier M (2006). Validation of the critical-care pain observation tool in adult patients. Am J Crit Care.

[21] Sessler CN, Gosnell MS, Grap MJ, Brophy GM, O'Neal PV, Keane KA (2002). The Richmond agitation-sedation scale: validity and reliability in adult intensive care unit patients. Am J Respir Crit Care Med.

[22] Ely EW, Inouye SK, Bernard GR, Gordon S, Francis J, May L (2001). Delirium in mechanically ventilated patients: validity and reliability of the confusion assessment method for the intensive care unit (CAM-ICU). JAMA..

[23] Ely EW, Margolin R, Francis J, May L, Truman B, Dittus R (2001). Evaluation of delirium in critically ill patients: validation of the confusion assessment method for the intensive care unit (CAM-ICU). Crit Care Med.

[24] Wang C, Wu Y, Yue P, Ely EW, Huang J, Yang X (2013). Delirium assessment using confusion assessment method for the intensive care unit in Chinese critically ill patients. J Crit Care.

[25] Zhao LH, Shi ZH, Chen GQ, Yin NN, Chen H, Yuan Y (2017). Use of dexmedetomidine for prophylactic analgesia and sedation in patients with delayed extubation after craniotomy: a randomized controlled trial. J Neurosurg Anesthesiol.

[26] Ritmala-Castren M, Lakanmaa RL, Virtanen I, Leino-Kilpi H (2014). Evaluating adult patients’ sleep: an integrative literature review in critical care. Scand J Caring Sci.

[27] Borson S, Scanlan J, Brush M, Vitaliano P, Dokmak A (2000). The Mini-cog: a cognitive ‘vital signs’ measure for dementia screening in multi-lingual elderly. Int J Geriatr Psychiatry.

[28] Althunian TA, de Boer A, Groenwold RHH, Klungel OH (2017). Defining the noninferiority margin and analysing noninferiority: an overview. Br J Clin Pharmacol.

[29] Wang S, Hong Y, Li S, Kuriyama A, Zhao Y, Hu J (2020). Effect of dexmedetomidine on delirium during sedation in adult patients in intensive care units: a systematic review and meta-analysis. J Clin Anesth.

[30] Liu X, Xiong J, Tang Y, Gong CC, Wang DF. Role of dexmedetomidine in the treatment of delirium in critically ill patients: a systematic review and meta-analysis. Minerva Anestesiol. 2020;87. 10.23736/S0375-9393.20.14492-4 33300321

[31] Dasenbrock HH, Yan SC, Chavakula V, Gormley WB, Smith TR, Claus EB (2017). Unplanned reoperation after craniotomy for tumor: a National Surgical Quality Improvement Program analysis. Neurosurgery..

[32] Patel MB, Bednarik J, Lee P, Shehabi Y, Salluh JI, Slooter AJ (2018). Delirium monitoring in neurocritically ill patients: a systematic review. Crit Care Med.

[33] Larsen LK, Frøkjaer VG, Nielsen JS, Skrobik Y, Winkler Y, Møller K (2019). Delirium assessment in neuro-critically ill patients: a validation study. Acta Anaesthesiol Scand.

[34] von Hofen-Hohloch J, Awissus C, Fischer MM, Michalski D, Rumpf JJ, Classen J (2020). Delirium screening in neurocritical care and stroke unit patients: a pilot study on the influence of neurological deficits on CAM-ICU and ICDSC outcome. Neurocrit Care.

[35] Casault C, Soo A, Lee CH, Couillard P, Niven D, Stelfox T (2021). Sedation strategy and ICU delirium: a multicentre, population-based propensity score-matched cohort study. BMJ Open.

[36] Lepouse C, Lautner CA, Liu L, Gomis P, Leon A (2006). Emergence delirium in adults in the post-anaesthesia care unit. Br J Anaesth.

